# Estimating and Characterizing COVID-19 Deaths, Puerto Rico, March–July 2020

**DOI:** 10.1177/0033354921991521

**Published:** 2021-02-17

**Authors:** Alejandro Azofeifa, Diana Valencia, Carmen J. Rodriguez, Maritza Cruz, Devin Hayes, Edén Montañez-Báez, Betzaida Tejada-Vera, Joshua E. Villafañe-Delgado, Jessica J. Cabrera, Miguel Valencia-Prado

**Affiliations:** 1 1242 82781 Centers for Disease Control and Prevention COVID-19 Emergency Response, Atlanta, GA, USA; 2 Puerto Rico Department of Health, San Juan, Puerto Rico

**Keywords:** COVID-19, mortality, excess deaths, surveillance, Puerto Rico

## Abstract

**Objectives:**

Using the Council of State and Territorial Epidemiologists (CSTE) classification guidelines, we characterized coronavirus disease 2019 (COVID-19)–associated confirmed and probable deaths in Puerto Rico during March–July 2020. We also estimated the total number of possible deaths due to COVID-19 in Puerto Rico during the same period.

**Methods:**

We described data on COVID-19–associated mortality, in which the lower bound was the sum of confirmed and probable COVID-19 deaths and the upper bound was excess mortality, estimated as the difference between observed deaths and average expected deaths. We obtained data from the Puerto Rico Department of Health COVID-19 Mortality Surveillance System, the Centers for Disease Control and Prevention’s National Electronic Disease Surveillance System Base System, and the National Center for Health Statistics.

**Results:**

During March–July 2020, 225 COVID-19–associated deaths were identified in Puerto Rico (119 confirmed deaths and 106 probable deaths). The median age of decedents was 73 (interquartile range, 59-83); 60 (26.7%) deaths occurred in the Metropolitana region, and 140 (62.2%) deaths occurred among men. Of the 225 decedents, 180 (83.6%) had been hospitalized and 93 (41.3%) had required mechanical ventilation. Influenza and pneumonia (48.0%), sepsis (28.9%), and respiratory failure (27.1%) were the most common conditions contributing to COVID-19 deaths based on death certificates. Based on excess mortality calculations, as many as 638 COVID-19–associated deaths could have occurred during the study period, up to 413 more COVID-19–associated deaths than originally reported.

**Conclusions:**

Including probable deaths per the CSTE guidelines and monitoring all-cause excess mortality can lead to a better estimation of COVID-19–associated deaths and serve as a model to enhance mortality surveillance in other US jurisdictions.

Puerto Rico is home to an estimated 3.6 million people.^1^ The Puerto Rico Department of Health (PRDoH) reported its first case of the novel coronavirus disease 2019 (COVID-19) caused by severe acute respiratory syndrome coronavirus 2 (SARS-CoV-2) on March 9, 2020, and the first COVID-19–associated death on March 17, 2020.^2^ As of July 31, 2020, the PRDoH had reported 17 872 confirmed COVID-19 cases (6543 by reverse transcriptase–polymerase chain reaction [RT-PCR], the molecular test for SARS-CoV-2 RNA, and 11 329 by a SARS-CoV-2 serology test).^2^

Estimating and characterizing COVID-19–associated deaths in the United States during the current pandemic has been challenging because of limited testing, variable testing sensitivity, misclassification of COVID-19–associated deaths, lack of completeness of COVID-19 case reports and surveillance forms, and reporting delays to local surveillance systems.^3,4^ Since April 5, 2020, the Council of State and Territorial Epidemiologists (CSTE) has recommended that all COVID-19 deaths be reported to local public health authorities.^5^ Based on the CSTE guidelines, a confirmed COVID-19 death case is defined as meeting confirmatory laboratory evidence (positive RT-PCR test result)^5^; a probable COVID-19 death case is defined as meeting 1 of the following criteria: (1) meets clinical criteria and epidemiological evidence without a positive RT-PCR test result, (2) meets presumptive laboratory evidence (serology test) and either clinical criteria or epidemiological evidence, or (3) meets vital records criteria (“a death certificate that lists COVID-19 disease or SARS-CoV-2 as a cause of death or significant condition contributing to death”) without a positive RT-PCR test result.^5^

In Puerto Rico, death from COVID-19 is a reportable event to the PRDoH Mortality Surveillance System (MSS). Accurate classification of COVID-19–associated deaths (confirmed and probable) might vary by reporting source and in some cases requires review by a multidisciplinary team at PRDoH. However, counting only confirmed and probable COVID-19–associated deaths might underestimate the number of deaths attributed to the pandemic. Deaths are not counted by MSS when they are not directly associated with SARS-CoV-2 infections, such as deaths that occur outside a health care setting or deaths that are misclassified by the attending health care provider, which may contribute to an underestimation of deaths associated with COVID-19. Estimating excess deaths can help explain the severity or burden of pandemics and public health emergencies.^6,7^ The objectives of our study were to characterize COVID-19–associated deaths in Puerto Rico during March–July 2020 (using CSTE guidelines) and estimate the range of COVID-19–associated mortality that may have been underestimated.

## Methods

### Puerto Rico Department of Health COVID-19 Mortality Surveillance System

PRDoH MSS is a passive surveillance system that was enhanced for the COVID-19 response by including additional data sources to verify information on deaths attributed to COVID-19. PRDoH MSS includes information on deaths from regional epidemiologists, vital statistics from physicians, and COVID-19 laboratory results (confirmed [RT-PCR molecular test for SARS-CoV-2 RNA] and probable [SARS-CoV-2 serology test])^8,9^ from Puerto Rico’s laboratory reporting system and the National Electronic Disease Surveillance System Base System.^10^ The National Electronic Disease Surveillance System Base System is an integrated information system developed by the Centers for Disease Control and Prevention (CDC) to help local, state, and territorial public health departments, such as PRDoH, manage notifiable disease data and send reports to CDC. Puerto Rico’s regional epidemiologists receive death information from all hospitals and collect and verify additional COVID-19 death data, including pending laboratory test results, requested by the central PRDoH MSS team. Regardless of where the COVID-19 death occurs (hospital, other health care facility, or home), death certificates are completed by physicians and sent to vital statistics. The PRDoH MSS central team links its data with data on COVID-19 deaths from vital statistics using the *International Classification of Diseases, Tenth Revision* code U07.1, COVID-19.^11^ The PRDoH MSS central team reconciles classification (confirmed or probable) from all reporting sources and reports the daily number of deaths (per date of death) due to COVID-19 into an electronic centralized system at the PRDoH. Deaths that were undefined or under investigation were reviewed by a multidisciplinary team when COVID-19 testing (confirmed and probable) and clinical or pathology information became available. Because public health surveillance data collected by PRDoH and analyzed in this article are not considered research, no institutional review board review was required.

### Statistical Analysis

Excess mortality is defined as the difference between observed deaths and average expected deaths in the same period as reported by CDC’s National Center for Health Statistics (NCHS).^12^ We calculated excess mortality using the Farrington surveillance algorithm applied to death data for Puerto Rico from 2013 through the present.^12^ Negative values, in which the observed count fell below the NCHS threshold, were set to zero^12^ and were not depicted. Details about NCHS methods and excess death data associated with COVID-19 can be found elsewhere.^12^ We calculated a range of deaths potentially associated with COVID-19. The lower bound of the range was the sum of confirmed and probable COVID-19 deaths reported by PRDoH during March 17–July 31, 2020 (calendar weeks 12-31). The upper bound of the range of COVID-19–associated deaths was the sum of excess deaths in Puerto Rico reported by NCHS during March 2–August 2, 2020 (calendar weeks 10-31).^2^ We calculated 95% CIs for the sum of excess deaths using the standard error of excess deaths for the study period. This range, the difference between the lower bound and the upper bound, represents an estimate of missed deaths that likely were associated with the COVID-19 pandemic. We also depicted the excess estimates for all cases of death excluding COVID-19 deaths to visualize the gap between excess mortality and COVID-19 deaths during the study period. We did not analyze data on underlying medical conditions, symptomology, or course of clinical illness and care received because this information was missing for 171 (76%) cases.

## Results

### Demographic Characteristics

As of July 31, 2020, PRDoH had officially reported 225 COVID-19 deaths (119 confirmed cases and 106 probable cases). Of the 225 COVID-19 deaths, 140 (62.2%) decedents were male (Table). The median age of people dying of COVID-19 in Puerto Rico was 73 (interquartile range, 59-83), and the highest percentage distributions by age ranges (in years) were 75-84 (27.1%), 65-74 (23.1%), 50-64 (19.1%), and ≥85 (18.7%). The highest percentage of deaths from COVID-19 occurred in the Metropolitana region (26.7%) followed by the Mayagüez region (15.6%) and the Ponce region (13.8%). Of the 225 COVID-19 deaths, 188 (83.6%) decedents had been hospitalized and 93 (41.3%) required mechanical ventilation. Based on death certificates, the most frequent conditions contributing to COVID-19 deaths were influenza and pneumonia (48.0%), sepsis (28.9%), and respiratory failure (27.1%).

**Table:** Number and percentage of deaths attributed to COVID-19 (119 confirmed, 106 probable), by age, jurisdiction (region), place of death, and underlying medical conditions contributing to deaths based on death certificate, Puerto Rico, March–July 2020^a^

Characteristic	Deaths attributed to COVID-19^b^
Age, median (IQR), y	73 (59-83)
Age group, y	
0-4	0
5-17	1 (0.4)
18-29	5 (2.2)
30-39	3 (1.3)
40-49	18 (8.0)
50-64	43 (19.1)
65-74	52 (23.1)
75-84	61 (27.1)
≥85	42 (18.7)
Sex	
Male	140 (62.2)
Female	85 (37.8)
Jurisdiction (region)^c^	
Aguadilla	10 (4.4)
Arecibo	22 (9.8)
Bayamón	27 (12.0)
Caguas	29 (12.9)
Fajardo	7 (3.1)
Mayagüez	35 (15.6)
Metropolitana	60 (26.7)
Ponce	31 (13.8)
Other^d^	4 (1.8)
Place of death	
Hospital	188 (83.6)
Outpatient (emergency department)	24 (10.7)
Home	7 (3.1)
Long-term care facility	4 (1.8)
Other^e^	2 (0.9)
Underlying medical conditions contributing to deaths based on death certificate^f^	
COVID-19	225 (100.0)
Respiratory diseases	
Influenza and pneumonia	108 (48.0)
Chronic lower respiratory diseases	11 (4.9)
Adult respiratory distress syndrome	32 (14.2)
Respiratory failure	61 (27.1)
Respiratory arrest	26 (11.6)
Other diseases of respiratory system	23 (10.2)
Circulatory diseases	
Hypertensive diseases	21 (9.3)
Ischemic heart disease	16 (7.1)
Cardiac arrest	26 (11.6)
Cardiac arrhythmia	7 (3.1)
Heart failure	13 (5.8)
Cerebrovascular diseases	6 (2.7)
Other diseases of the circulatory system	3 (1.3)
Sepsis	65 (28.9)
Malignant neoplasm	9 (4.0)
Diabetes	23 (10.2)
Obesity	2 (0.9)
Alzheimer disease	6 (2.7)
Vascular and unspecified dementia	3 (1.3)
Renal failure	23 (10.2)
Intentional and unintentional injury, poisoning, and other adverse events	0
All other conditions and causes (residual)	48 (21.3)
Missing	26 (11.6)

Abbreviations: COVID-19, coronavirus disease 2019; IQR, interquartile range.

^a^Data source: Puerto Rico Department of Health Mortality Surveillance System.

^b^All values are number (percentage) except where indicated. Percentages may not total to 100 because of rounding.

^c^Names of municipalities in Puerto Rico within each region. Aguadilla: Aguada, Aguadilla, Isabela, Moca, and San Sebastián; Arecibo: Arecibo, Barceloneta, Camuy, Ciales, Florida, Hatillo, Lares, Manatí, Morovis, Quebradillas, Utuado, and Vega Baja; Bayamón: Barranquitas, Bayamón, Cataño, Comerío, Corozal, Dorado, Naranjito, Orocovis, Toa Alta, Toa Baja, and Vega Alta; Caguas: Aguas Buenas, Aibonito, Caguas, Cayey, Cidra, Gurabo, Humacao, Juncos, Las Piedras, Maunabo, Naguabo, San Lorenzo, and Yabucoa; Fajardo: Ceiba, Culebra, Fajardo, Luquillo, Río Grande, and Vieques; Mayagüez: Añasco, Cabo Rojo, Hormigueros, Lajas, Las Marías, Maricao, Mayagüez, Rincón, Sabana Grande, and San Germán; Metropolitana: Canóvanas, Carolina, Guaynabo, Loíza, San Juan, and Trujillo Alto; Ponce: Adjuntas, Arroyo, Coamo, Guánica, Guayama, Guayanilla, Jayuya, Juana Díaz, Patillas, Peñuelas, Ponce, Salinas, Santa Isabel, Villalba, and Yauco.

^d^Other is defined as people who are from another US state or another country.

^e^Other includes other places such as correctional facilities or unknown places.

^f^Conditions contributing to deaths were identified by mention on death certificate. Numbers and percentages do not total to the number of cases or 100% because these data are not mutually exclusive. Data during the study period are incomplete because of the lag between when the death occurred and when the death certificate is completed, submitted, and processed to the Puerto Rico Department of Health.

### Excess Deaths Associated With COVID-19

During March–July 2020, a total of 863 deaths were reported to NCHS and PRDoH; of these deaths, 638 (95% CI, 625-651) were found to be in excess of the expected baseline. Included in the 638 deaths were the reported 119 confirmed COVID-19 cases and 106 probable COVID-19 cases, leaving as many as 413 possible additional COVID-19–associated deaths that were assigned another cause of death (Figure).

**Figure:**
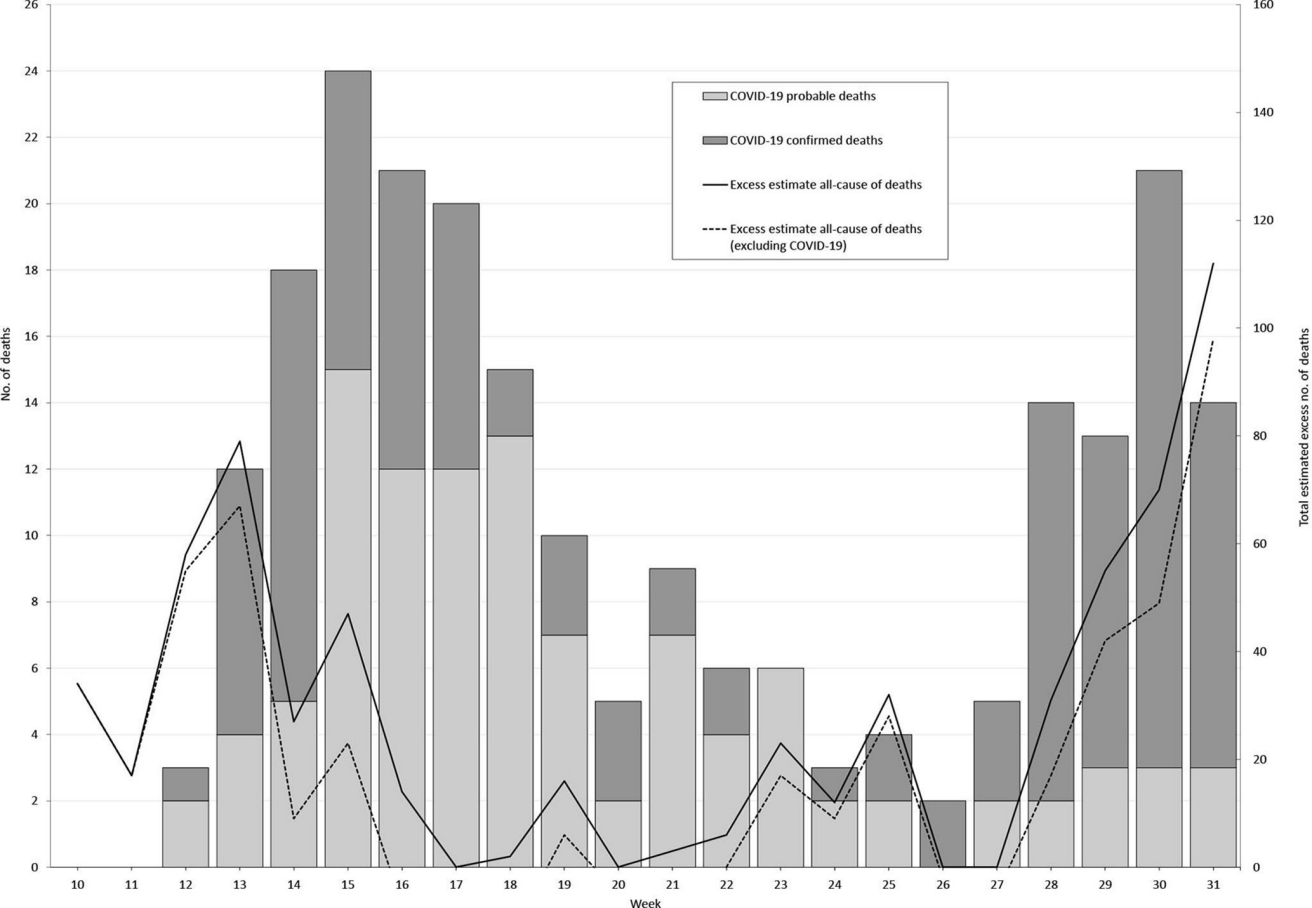
Number of laboratory-confirmed (n = 119) and probable (n = 106) COVID-19–associated deaths and total estimated excess deaths, Puerto Rico, March–July 2020. A death case is a person meeting confirmatory laboratory evidence (reverse transcriptase–polymerase chain reaction [RT-PCR] molecular test) during March–July 2020 reported by the Puerto Rico Department of Health.^4^ A death case is a person meeting 1 of the following criteria: (1) meets clinical criteria and epidemiological evidence without a positive RT-PCR molecular test result, (2) meets a presumptive laboratory evidence (serology antibody test) and either clinical criteria or epidemiological evidence, or (3) meets vital records criteria (“a death certificate that lists COVID-19 disease or SARS-CoV-2 [severe acute respiratory syndrome coronavirus 2] as a cause of death or significant condition contributing to death”) without a positive RT-PCR molecular test^4^ during March 17–July 31, 2020 (calendar weeks 12-31) reported by the Puerto Rico Department of Health.^4^ Total excess all-cause deaths reported by the National Center for Health Statistics were estimated using the Farrington surveillance algorithm with the data from 2013 through July 31, 2020, as the difference between observed and expected deaths during March 2–August 2, 2020 (calendar weeks 10-31). Negative excess death estimates were set to zero and are not depicted.^10^ Abbreviation: COVID-19, coronavirus disease 2019.

## Discussion

Since the early stages of the COVID-19 pandemic, monitoring and estimating COVID-19–associated deaths in the United States has been challenging. Public health officials have raised concerns about estimating the “true” death toll resulting from COVID-19.^3^ Considering excess COVID-19–associated mortality might improve our understanding of how many deaths are attributed to the pandemic. Although these methods are being used in the surveillance of COVID-19 mortality,^3,12-14^ the results of our analysis suggest that many COVID-19–associated deaths in Puerto Rico during the pandemic were missed—approximately 2 times higher than originally reported—during the study period; as many as 413 COVID-19–associated deaths might have been uncounted. Previous national studies support that deaths from COVID-19 are likely underreported by local surveillance systems.^3,4,14^ Increased COVID-19 testing and completeness of death reporting under local surveillance could enhance the understanding of the number of deaths caused by this evolving disease and pandemic. We suspect that reported deaths associated with COVID-19 could increase substantially as testing and local surveillance systems improve case ascertainment and classification.

Our study results also reaffirm the importance of using CSTE COVID-19 death case definitions in reporting deaths. Of the 225 COVID-19 cases in Puerto Rico, almost half were probable cases. The CSTE case definition was also used in New York City COVID-19 mortality surveillance, with 5048 probable cases added to 13 831 laboratory-confirmed COVID-19 deaths.^13^ The inclusion of probable cases is particularly important because, at the time of the study, Puerto Rico had the highest number of probable COVID-19 cases in the country. This new pathogen has placed new and unique demands on local public health surveillance systems and infrastructures everywhere.^15^ Therefore, complementing data with NCHS data on excess deaths (measuring all causes of death) is a more sensitive measure than only counting COVID-19 confirmed and probable deaths.

Based on our reported data, deaths due to COVID-19 in Puerto Rico are particularly affecting older people and people living in the most populated regions. Data show that most people die in health care settings (hospital or emergency department). Similar to national data, early local pandemic data show that the number of hospital visits was lower than usual, perhaps because of strict public health mitigation measures related to COVID-19.^16,17^ We hypothesized that people in Puerto Rico might have delayed or avoided medical care because of concerns about exposure to COVID-19. Delay or avoidance might have exacerbated uncontrolled chronic conditions or circulating infections, as shown by the conditions that contributed to death indicated on death certificates. Local public health officials should emphasize the importance of attending regular medical appointments to enhance the public’s awareness and action on medical care. Public health actions could be strengthened by tailoring public messaging to populations that may require special assistance to attend their medical appointments.

### Limitations

This study had several limitations. First, both incomplete and missing data on COVID-19 deaths in the PRDoH MSS database limited our ability to better understand contributing mortality factors or course of illness among the population; national data have had similar limitations (almost 60% of missing mortality data).^4^ Second, the fragmented surveillance systems for the COVID-19 response in Puerto Rico likely underestimated COVID-19 death counts. However, the underestimation of deaths could be a common pattern during the current COVID-19 response in many jurisdictions in the United States and elsewhere.^3,18-20^ Third, excess mortality trend data do not align with the PRDoH epidemiological curve depicted for COVID-19 deaths, as previously reported in COVID-19 analyses.^3,13,14^ This misalignment may have resulted from the lag between the occurrence of the death and the completion and submission of the death certificate by local public health officials to NCHS. Per NCHS, such data lags can range from 1 to 8 weeks.^11^ Therefore, use of local data to depict mortality trends might buffer possible differences in national reporting delays.^21^ Fourth, conditions contributing to death based on death certificates might be incomplete or under investigation. Therefore, our results should be interpreted with caution, as more detailed analyses are needed. Finally, overattributing deaths directly to COVID-19 during the current pandemic could underestimate data on mortality from other causes. Therefore, a more detailed analysis of deaths, considering delay or access to care, might be warranted.

## Conclusions

Puerto Rico, as with many other US jurisdictions, has been affected by the COVID-19 pandemic. Similar to a previous postdisaster study conducted on the island,^7^ all-cause excess mortality proved to be a useful public health tool to monitor the current COVID-19 pandemic. Only counting laboratory-confirmed or probable COVID-19–associated deaths likely underestimates the true number of deaths associated with the pandemic. To better understand underlying medical conditions that contribute to deaths during the pandemic, more complete investigations, including the completion of CDC’s standardized case-report form,^22^ with medical record abstraction and including additional data sources, could be enhanced. Given the variability of reporting sources and laboratory COVID-19 test results for deaths, PRDoH MSS could consider expanding its passive surveillance efforts to an active or hybrid surveillance to increase death case ascertainment and completeness. Ascertainment of deaths during a pandemic could also take into consideration public health restrictions (eg, lockdown measures, travel restrictions, and total or partial interruption of public transportation) and how they might have affected a person’s ability to attend regular medical appointments for chronic or new conditions. Public health officials could consider enhancing the public’s awareness of the importance of attending regularly scheduled medical appointments to avoid exacerbation of chronic or acute illness. Enhanced mortality surveillance can provide vital information to help monitor the severity and progression of the COVID-19 pandemic by estimating deaths directly and indirectly associated with COVID-19. Health department monitoring of all causes of excess death during the COVID-19 pandemic could help guide public health decision making, messaging, and interventions to help prevent additional deaths.
